# Tissue Transglutaminase but Not Microbial Transglutaminase Is Inhibited by Exogenous Oxidative Substances in Celiac Disease

**DOI:** 10.3390/ijms23042248

**Published:** 2022-02-17

**Authors:** Sebastian Stricker, Silvia Rudloff, Jan De Laffolie, Klaus-Peter Zimmer

**Affiliations:** 1 Department of Pediatrics, Justus-Liebig-University Giessen, 35392 Giessen, Germany; silvia.rudloff@ernaehrung.uni-giessen.de (S.R.); jan.delaffolie@paediat.med.uni-giessen.de (J.D.L.); klaus-peter.zimmer@paediat.med.uni-giessen.de (K.-P.Z.); 2 Institute of Nutritional Science, Justus-Liebig-University Giessen, 35392 Giessen, Germany

**Keywords:** celiac disease, transglutaminase 2, microbial transglutaminase, TG2 inhibitor, ERp57

## Abstract

Enzymatic modification of gliadin peptides by human transglutaminase 2 (TG2) is a central step in celiac disease (CD) pathogenesis. Microbial transglutaminase (mTG) mimics the enzymatic function of TG2 and might play a role in CD. TG2 is inhibited by endogenous oxidative endoplasmic reticulum-resident protein 57 (ERp57), but data about mTG are lacking. We investigated the localization of ERp57 in duodenal biopsies and examined inhibition of TG2, and mTG by competitive, and oxidative molecules. Localization of ERp57 was investigated in duodenal biopsies from CD, and control patients by electron microcopy. Inhibition of TG2 and mTG was analyzed on an in vitro level using a photometric assay. ERp57 was observed within the lamina propria and its abundance within the endoplasmic reticulum (ER) was reduced in CD patients. TG2 was oxidatively inhibited by up to 95% by PX12 (*p* < 0.001) and L-cystine (*p* < 0.001), whereas mTG remained unaffected. The reduced presence of ERp57 within the ER of CD biopsies suggests a regulatory function of this protein within CD pathogenesis. PX12 and L-cystine oxidatively inhibit TG2 and might serve as treatment options in CD. mTG is poorly regulated and could contribute to the accumulation of immunogenic peptides within the gut with potential pathogenic effects.

## 1. Introduction

Celiac Disease (CD) is a frequent chronic inflammatory disorder triggered by the ingestion of gliadin and related prolamins in genetically predisposed individuals carrying the HLA DQ2/8 genotype [1]. One central step in its pathogenesis is de- and transamidation of gliadin peptides by tissue transglutaminase (TG2), the recognized autoantigen of the disease [2]. This enzymatic modification markedly increases the immunogenic properties of those gliadin peptides, and is a prerequisite for any subsequent antigen presentation resulting in a TH_1_-mediated immune response [1,2,3,4]. In addition to TG2, microbial transglutaminase (mTG) was recently suggested to take part in CD pathogenesis, as it is also able to de- and transamidate the same glutamine residues within gliadin peptides, rendering them immunogenic for gluten-specific T cells [5,6,7]. In the food industry, mTG is used as a technological aid to improve the textural properties of various of food products without the need for declaration in the European Union [8,9]. Though enzymatic activity of mTG is abolished during food processing, its usage could create immunogenic epitopes and complexes within food stuffs. Further, mTG may be released in an active form by our intestinal microbiota (e.g., streptomycetes), contributing additional transglutaminase activity to the gut lumen [10]. 

Recently, it was shown that TG2 activity underlies regulatory processes in the extracellular matrix by means of a redox reaction of a disulfide bond [11]. The endoplasmic reticulum-resident protein 57 (ERp57) exerts an inhibitory effect on TG2 by oxidation, whereas Thioredoxin-1 (TRX) promotes TG2 activity by reduction [12,13,14]. ERp57 is predominantly considered an intracellular protein and plays a crucial role in cross presentation, where it contributes to the assembly of MHC (major histocompatibility complex) class I [15]. Cross presentation describes the presentation of exogenous antigens (e.g., gliadin peptides) via MHC I molecules to CD8^+^ T cells, a mechanism which is not only essential for the immune response against viruses, but also for tolerance against self- and oral antigens [16,17,18,19,20,21]. The mechanism takes place in the ER of professional (dendritic cells) and non-professional (e.g., enterocytes) antigen presenting cells and might contribute to CD8^+^ T cell-mediated tissue destruction in CD pathogenesis [15,22]. One special cell type called RACE (Rapid Antigen Uptake into the Cytosol of Enterocytes) seems to be predestinated for cross presentation, as these cells show a dilated ER and a high uptake of exogenous antigens into this compartment [23]. 

The aim of this study was to investigate the intra- and extracellular distributions of TG2 inhibitory ERp57 in intestinal biopsies of CD patients and controls, and to test the effects of competitive and oxidative inhibitors on TG2 and mTG activity to screen for new potential therapeutic agents in CD.

## 2. Results

### 2.1. Localization of ERp57 within Intra- and Extracellular Compartments of Human Duodenal Epithelium

**Figure 1:**
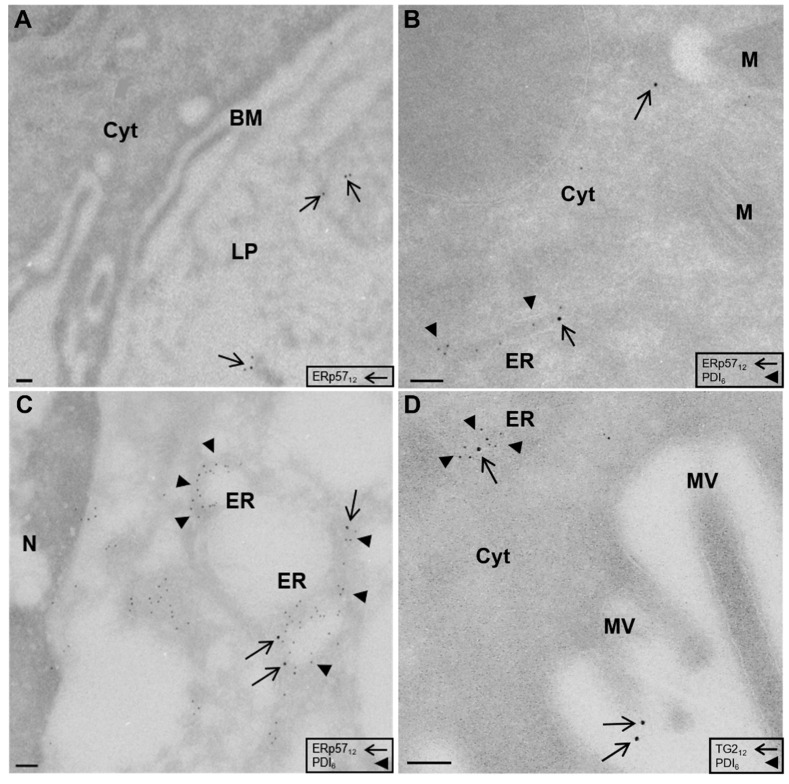
Localization of endoplasmic reticulum-resident protein 57 (ERp57) in the ER and the lamina propria of duodenal biopsies. (**A**) Electron microscopical localization of ERp57 (arrows) within intestinal lamina propria of human duodenal epithelium (CD patient, magnification × 10,200). (**B**) Presence of ERp57 (arrows) within the ER (labelled by PDI, arrowheads) and the cytosol of an enterocyte (NCD patient, magnification × 17,700). (**C**) Detection of ERp57 (arrows) within the dilated perinuclear ER (arrowheads) of RACE characterized by electron lucent cytosol (NCD patient, magnification × 17,700). (**D**) TG2 (arrow) within the ER (arrowhead) and at the apical membrane of an enterocyte (CD patient, magnification × 17,700). BM, basolateral membrane; Cyt, cytosol; LP, lamina propria; M, mitochondria; MV, microvilli; N, nucleus. Scale 100 nM.

As recent evidence has shown that ERp57 inhibits extracellular TG2 activity by oxidation, we determined its localization and distribution within human intestinal biopsies of CD patients and controls. ERp57 is a key player in cross presentation, an immune reaction that initiates within the ER, so that we specifically wanted to analyze its abundance within this compartment. For this purpose, duodenal biopsies of CD and non-celiac disease (NCD) patients on gluten-containing diet were obtained and investigated using the cryo-immunogold method. Lack of labelling within goblet cell granules served as negative control to ensure specificity of the used ERp57 antibody. Using this method, we could observe significant amounts of ERp57 within the intestinal lamina propria (Figure 1A). In addition to its extracellular presence, we observed relevant amounts of ERp57 in intracellular compartments, including vacuoles, the endoplasmic reticulum (ER), and the cytosol. Only minor amounts of ERp57 were abundant at the apical and basolateral membrane (Figure 1B,C).

### 2.2. High Amounts of ERp57 Are Localized in the ER of RACE

**Figure 2:**
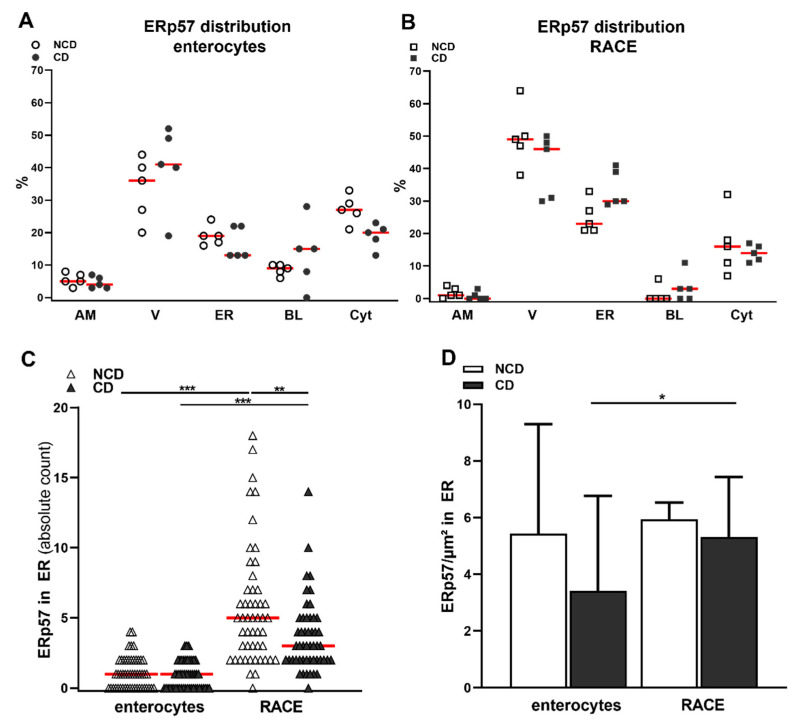
Higher abundance of ERp57 within the ER of RACE and control patients. (**A**) High amounts of ERp57 within vacuoles, the ER and the cytosol of enterocytes. Only small proportions were detected at the apical and basolateral membrane. (**B**) High amounts of ERp57 within vacuoles, the ER and the cytosol of RACE. Higher detection of ERp57 within the ER of RACE displayed as absolute count of gold particles labelling ERp57. Reduced detection of ERp57 within the ER of RACE in CD biopsies. (**C**) ERp57 concentration trended to be higher in the ER of RACE compared to enterocytes and in the NCD group compared to CD group. A/B) Graphs display the values from each individual, red line = median. (**C**) Red line = median. (**D**) Data are shown as median and 97% CI, *n* = 50 in each group. * *p* < 0.05, ** *p* < 0.01, *** *p* < 0.001.

In enterocytes, the majority of ERp57 was observed within vacuoles (33 ± 10% in NCD, 40 ± 13% in CD), but we could also detect significant amounts within the endoplasmic reticulum (19 ± 3% in NCD, 16 ± 5% in CD) and the cytosol (Figure 1B and Figure 2A, Table 1). There were no significant differences in the epithelial distribution pattern of ERp57 between NCD and CD patients. The specific epithelial cell type RACE was observed in all investigated samples. RACE showed higher amounts of ERp57 per cell compared to enterocytes (Table 1). In RACE, ERp57 was most prominent in vacuoles, the ER and the cytosol. However, relative abundance of ERp57 within the ER was significantly higher in RACE compared to enterocytes (18 ± 4% vs 30 ± 7%, *p* < 0.001, Figure 1B,C and Table 1).

Corresponding to this, we observed reduced labelling of this protein at the apical membrane, the basolateral membrane and the cytosol of RACE (Figure 2B and Table 1). Using a polyclonal antibody directed against human TG2, we detected TG2 in the intestinal lamina propria as could be expected. Further, TG2 was present in intracellular compartments including the ER, the apical membrane and the cytosol of enterocytes (D).

RACE show a higher amount of ERp57 as observed by an increased number of gold particles per cell. Increased volume of the ER in RACE compared to enterocytes, but without a difference between CD and NCD patients. Higher presence of ERp57 within the ER of RACE compared to enterocytes (CD and NCD samples combined). IQR, interquartile range.

### 2.3. Reduced Abundance of TG2 Inhibitory ERp57 within the ER of CD Biopsies

Our results displayed several intracellular compartments containing high amounts of ERp57. The ER was one of the most prominent compartments and is associated with the mechanism of cross presentation, so that we further quantified the abundance of ERp57 within this compartment. The method to evaluate organelle circumference was adapted from Griffiths et al. and revealed a more than fivefold expansion of the ER within RACE (Table 1) [24].

There was no difference in ER extent between NCD and CD groups in both cell types. The absolute amount of ERp57 within the ER of RACE was significantly higher compared to enterocytes (NCD group: median 1, IQR 2 vs. median 5, IQR 5, *p* < 0.001, CD group: median 1, IQR 2 vs. median 3, IQR 3, *p* < 0.001, Figure 2C), confirming our previous results. In addition, there was a significantly higher amount of ERp57 in the ER of RACE of control patients compared to CD patients (*p* < 0.01, Figure 2C). 

The concentration of ERp57 in the ER as determined by calculating the labelling density appeared to be higher in RACE compared to enterocytes. This, however, was only statistically significant in the CD group (NCD group: median 5, IQR 12 GP/µm^2^ vs. median 6, IQR 5 GP/µm^2^, *p* = 0.5; CD group: median 3, IQR 9 vs. median 5, IQR 7, *p* < 0.05, Figure 2E). Further, ERp57 concentration trended to be higher in the NCD group in enterocytes and RACE (Figure 2D). 

The reduced amount of ERp57 within the ER of CD patients might indicate a reduction in its expression in CD pathogenesis. Further, the high amount of ERp57 found in RACE and specifically within the ER of RACE could further suggest a protective role of this cell type in CD.

### 2.4. TG2 Is Regulated by Endogenous Oxidative Molecules

**Figure 3:**
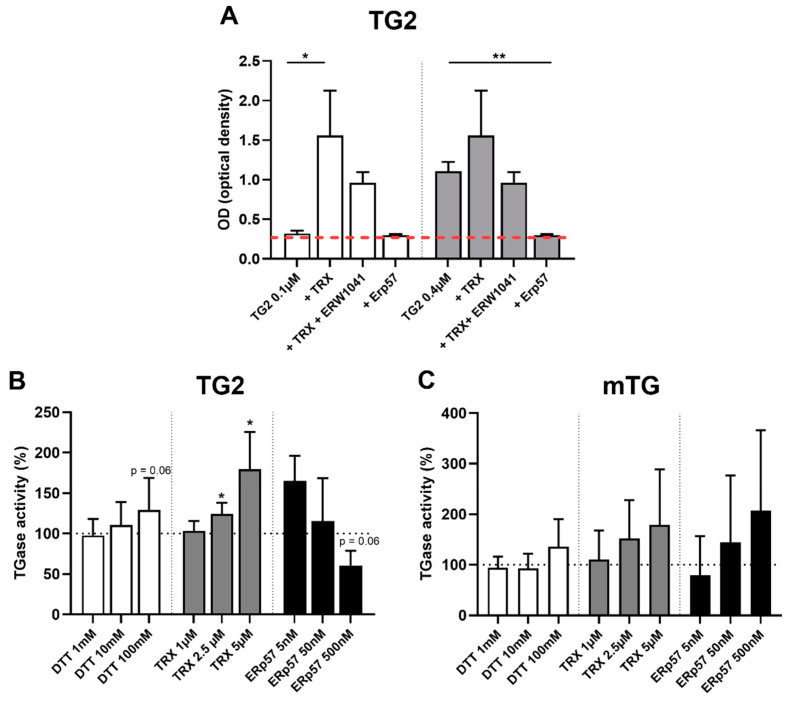
Oxidoreductive regulation of transglutaminase 2 (TG2) by endogenous thioredoxin-1 (TRX) and ERp57. (**A**) Two different doses of TG2 were immobilized onto fibronectin-coated 96 well plates. Significant stimulation of low levels of TG2 (0.1 µM) by TRX. Coincubation with ERW1041 at 200-fold molar excess reduced this effect by trend. At higher doses of TG2 (0.4 µM), stimulation by TRX (4 µM) was less pronounced, but enzymatic activity could be reduced up to the background level via oxidation by ERp57. (**B**) 0.1 µM TG2 was immobilized onto tissue culture-treated 96 well plates. Reduction by dithiothreitol (DTT) resulted in a slightly, not statistically significant enhanced TG2 activity. TRX significantly raised TG2 activity in a dose-dependent manner. Oxidation of TG2 by ERp57 trended to reduce TG2 activity. (**C**) mTG activity was not significantly influenced by DTT, TRX and ERp57. B) Red line = background signal. B/C) Black dashed line = control condition. Data are shown as mean ± standard deviation. * *p* < 0.05, ** *p* < 0.01.

Next, we investigated the effects of endogenous oxidative molecules, including ERp57, on TG2 and mTG enzyme activity using an in vitro assay. At low doses of TG2, transamidation could be significantly increased (more than fivefold) by treatment with TRX (1 µM, 0.3 ± 0.03 vs 1.6 ± 0.6, *p* < 0.05), an effect that could be reduced by trend using the TG2-specific competitive inhibitor ERW1041 (1.6 ± 0.6 vs 1.0 ± 0.1, *p* > 0.05, Figure 3A). At increased doses, where basic TG2 activity was much higher, the stimulatory effect of TRX was less pronounced and not statistically significant. Treatment of TG2 with ERp57 reduced transamidation activity up to the background level (2 µM, 1.1 ± 0.1 vs. 0.3 ± 0.01, *p* < 0.01, Figure 3A). 

To further investigate those observations, we performed concentration series. The substance DTT was used as positive control, but only showed stimulation of TG2 activity by trend (100 mM DTT 129 ± 40%, *p* = 0.06, Figure 3B). Reduction of TG2 by TRX markedly increased TG2 activity at doses of 2.5 µM (124 ± 14%, *p* < 0.05) and 5 µM (180 ± 46%, *p* < 0.05), whereas ERp57 treatment trended to reduce crosslinking at a dose of 0.5 µM (61 ± 18%, *p* = 0.06) (Figure 3B). Next, we addressed the question of whether those oxidative regulatory mechanisms were TG2-specific, or whether mTG was regulated as well. mTG showed comparable crosslinking at a dose of 100 nM, but we observed much more interassay variability of its enzymatic function compared to TG2 (TG2 activity: 0.62 ± 0.07, mTG activity 0.47 ± 0.28). There were no significant dose-dependent effects of the tested endogenous oxidative molecules TRX and ERp57 and the control substance DTT on mTG activity, indicating a rather unique and specific oxidative regulation of the human enzyme (Figure 3C).

### 2.5. Selective Inhibition of TG2 and mTG by Exogenous Competitive Inhibitors

**Figure 4:**
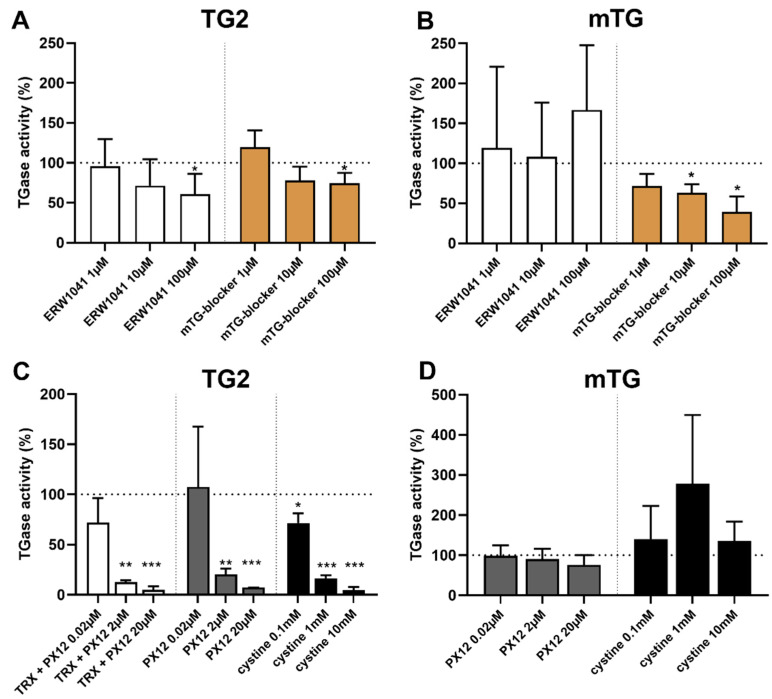
Effect of exogenous competitive and oxidative inhibitors on transglutaminases. (**A**) The competitive inhibitor ERW1041 selectively reduced TG2 activity at a dose of 100 µM. The competitive mTG-blocker only showed minor inhibition of TG2 at a dose of 100 µM. (**B**) mTG activity was not decreased by the competitive TG2 inhibitor ERW1041. Competitive mTG-blocker showed a strong and dose-dependent inhibition of mTG-mediated. (**C**) TRX was used at a 50-fold molar excess. Thioredoxin-mediated activation of TG2 could be abolished by PX12 at doses 2 µM and 20 µM. PX12 displayed a direct inhibitory effect on TG2 activity at 2 µM and 20 µM. L-cystine significantly reduced TG2 activity at all doses tested. (**D**) PX12 and L-cystine did not show any significant dose-dependent effect on microbial transglutaminase (mTG) activity. Data are shown as mean ± standard deviation.* *p* < 0.05, ** *p* < 0.01, *** *p* < 0.001.

Next, we investigated the effect of two competitive inhibitors on both transglutaminases. The competitive TG2 inhibitor ERW1041 displayed a dose-dependent inhibition of TG2 starting at a 100-fold (10 µM, 72 ± 33%, *p* = 0.08) excess and decreasing crosslinking of 5BP by about 40% at a 1,000-fold excess (100 µM, 61 ± 26%, *p* < 0.05, Figure 4A). The effect of the competitive mTG-blocker was less prominent, reducing TG2 activity by 25% at a 1000-fold excess (100 µM, 75 ± 12%, *p* < 0.05). mTG activity was not influenced by ERW1041 underlining the specificity of this competitive inhibitor. mTG-blocker showed strong inhibitory effect at a 10 to 1000-fold excess, reducing mTG activity by 28% (1 µM, *p* = 0.08), 37% (10 µM, *p* < 0.05) and 61% (100 µM, *p* < 0.05, Figure 4B).

### 2.6. Oxidative Inhibition of TG2 by Exogenous PX12 and Cystine

As we showed that TG2 is regulated by endogenous oxidative molecules, we examined the effect of exogenous oxidative substances on transglutaminase activity in the next step. We applied a recognized inhibitor of TRX in order to reduce the stimulatory effect of TRX on TG2.

First, we could show that a fourfold excess of PX12 (20 µM) abolishes TRX (5 µM)-mediated activation of TG2 (50 nM, 180 ± 46% vs. 40 ± 15%, *p* < 0.01, data not shown). This effect was confirmed using concentration series of PX12, where higher doses of PX12 markedly reduced TG2 activity below basic enzyme activity (TRX 2.5 µM, PX12 2.0 µM: 13 ± 2%, *p* < 0.01, PX12 20 µM: 5 ± 2%, *p* < 0.001, Figure 4C). This observation indicated a direct inhibitory effect, so we tested direct inhibition of TG2 by PX12 in the next step. Here, we demonstrated a dose-dependent reduction in TG2 mediated crosslinking at doses of 2 µM (20 ± 6%, *p* < 0.01) and 20 µM (7 ± 0.1%, *p* < 0.001, Figure 4C). Further studies using PX 12 at doses between 0.05 and 8 µM were conducted, and an IC_50_ of 0.7 µM (TG2 100 nM) was calculated using a variable slope model. 

These results prompted us to investigate another small oxidative molecule, L-cystine. This substance significantly reduced TG2 activity at doses of 0.1 mM (72 ± 9%, *p* < 0.05), 1 mM (16 ± 3%, *p* < 0.001), and 10 mM (5 ± 3%, *p* < 0.001, Figure 4C). Again, we calculated an IC_50_ of 0.14 mM by further assays using doses between 1 µM and 10 mM (TG2 100 nM). To investigate whether those effects were TG2-specific, we tested the influence of PX12 and L-cystine on mTG activity (Figure 4D). mTG-mediated crosslinking remained unaffected by both molecules, indicating a specific oxidative regulation of human TG2.

## 3. Discussion

Although central aspects of CD pathogenesis such as enzymatic modification of gliadin peptides by TG2 are well understood, it is still open as to why only a small proportion of those who are genetically predisposed suffer from the disease [25]. As de- and transamidation of gliadin peptides appear to be a major event, one can assume that dysregulation of TG2 activity, or additional transglutaminase activity e.g., by mTG could play a role in CD pathogenesis and might reveal potential therapeutic targets [26]. 

**Figure 5:**
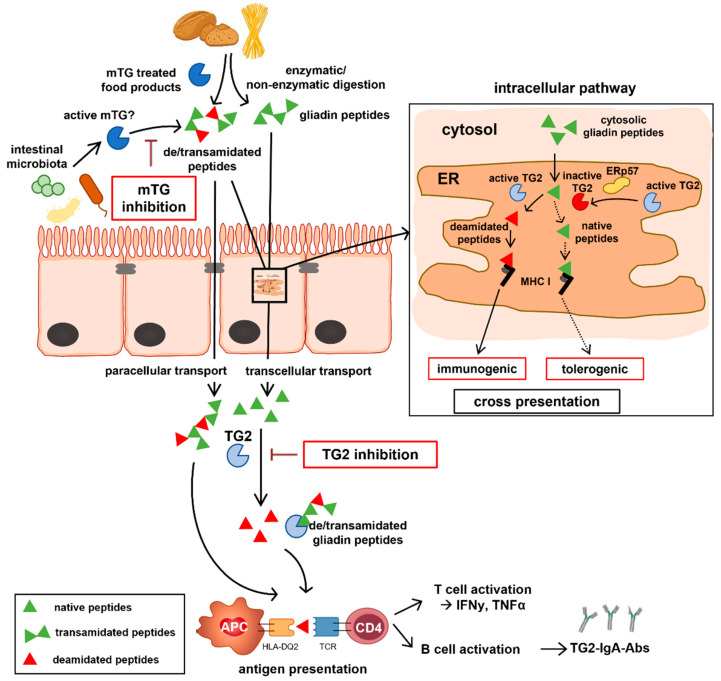
Role of intra/extracellular TG2 and mTG within CD pathogenesis. Gliadin peptides are resistant to enzymatic and non-enzymatic digestion and reach the intestinal lamina propria by trans- and paracellular routes. Enzymatic modification of gliadin peptides by TG2 increases their antigenicity and is a prerequisite for MHC class II mediated antigen presentation resulting in T and B cell activation. mTG used in food production can de- and/or transamidate gliadin peptides and create neo-epitopes, whereas active mTG released from the intestinal microbiota could modify peptides within the gut lumen. At the intracellular pathway, gliadin peptides reach the ER by phagosome-to-cytosol pathway. Within the ER, deamidation of gliadin peptides by active TG2 and mTG might occur, an effect that could be reduced via inhibition of TG2 (but not of mTG) by ERp57. The corresponding deamidated peptides might serve as substrate for MHC class I mediated immunogenic cross presentation ending up by the action of intraepithelial lymphocytes in villous atrophy, whereas native peptides could potentially induce tolerance via cross presentation.

Recent evidence indicated that the endogenous protein ERp57 inhibits TG2 activity in the extracellular matrix by oxidation, which prompted us to investigate the intra-, and extracellular presence of this molecule within human intestinal biopsies of CD patients and controls. We found significant amounts of ERp57 within the duodenal lamina propria, where TG2-mediated deamidation of gliadin peptides is supposed to take place. More-over, we detected ERp57 in intracellular compartments including the ER, the cytosol and vacuoles. As significant amounts of ERp57 were found within the ER of both enterocytes and RACE, we further quantified the abundance of this protein within this organelle. Strikingly, we observed more ERp57 in RACE and in particular within the ER of this specific cell type. In CD biopsies, we found reduced levels of ERp57 within the ER of RACE and enterocytes. Further, we displayed intra- and extracellular localization of TG2 and ERp57 suggesting a possible interaction between those molecules. Our results indicate a potential regulatory role of ERp57 on TG2 activity within the ER in CD pathogenesis. One could hypothesize that reduced presence of the endogenous TG2 inhibitor ERp57 within the ER of CD epithelium might favor the cross presentation of modified immunogenic gliadin peptides resulting in CD8^+^ T cell activation and epithelial destruction [18,27]. Higher amounts of ERp57 within the ER of the healthy gut epithelium could otherwise inhibit deamidation of gliadin peptides and thus favor cross presentation of native peptides, which could promote oral tolerance (Figure 5).

After evaluation of the intra- and extracellular distribution of the endogenous TG2 inhibitor ERp57, we tested endogenous and exogenous molecules to identify potential novel therapeutic options in CD. For this purpose, we used an in vitro transamidation assay as a surrogate of transglutaminase function. We could confirm the regulatory function of the endogenous oxidative molecules ERp57 and TRX on TG2 [11,12]. Finally, we addressed the question of whether exogenous competitive and oxidative compounds are able to inhibit TG2 and mTG. 

We investigated the impact of two exogenous oxidative inhibitors obtaining auspicious results for the small molecule PX12 and L-cystine [28,29]. PX12 which was considered as TRX inhibitor before, showed a strong, direct inhibitory effect on TG2 activity with an IC_50_ of 0.7µM. This substance has already been tested as an anti-tumor drug and has passed first clinical trials where it was tolerated up to a dose of 226 mg/m² by 3-h infusion [29,30]. L-cystine was used as supplement in clinical trials (700 mg per day) that demonstrated reduced postoperative inflammation in gastric cancer patients and reduced neuropathy in colorectal cancer patients under chemotherapy [31,32]. The in vitro data obtained in our study show that both molecules PX12 and L-cystine are potent inhibitors of TG2. Further research concerning inhibition of TG2 by oxidative molecules is clearly needed, and could augment the portfolio of therapy candidates in CD apart from competitive inhibitors [33].

In contrast to TG2, mTG activity was not affected by any oxidative molecule and the specific competitive TG2 inhibitor ERW1041. This inhibitor works by irreversible binding of the cysteine residue within the active center of TG2. The primary structures of TG2 and mTG do not show similarity, but both their active sites are composed by cysteine, histidine and aspartate. The fact that ERW1041 does not bind to the cysteine residue of mTG, further underlines the specificity of this inhibitor to TG2. The observation that oxidative substances do not impair mTG activity is explained by the fact that the primary structure of mTG does not contain further cysteine residues that could contribute to disulfide bond formation. 

The only substance that displayed reliable dose-dependent inhibition of the enzyme was the mTG-blocker, which is claimed to inhibit mTG by the same mechanism as ERW1041 blocks TG2. Our data indicate that mTG activity is less regulated than TG2. Keeping in mind that mTG works calcium-independent and has a much lower substrate specificity compared to TG2, the presence of active mTG within the gut lumen (e.g., as a product of the microbiota) might provide a multitude of neo-epitopes and increase the load of immunogenic peptides within the intestinal epithelium and the lamina propria [10,34,35,36]. Further, the ingestion of modified proteins by mTG-treated food products might contribute to this effect as well. mTG is widely used in the food industry to improve textural properties of a great variety of food products (even gluten-free products) without a need for declaration in the European Union [9]. Recent studies have indicated that mTG exerts trans- and deamidation and is, in contrast to other transglutaminases, able to create immunogenic gliadin peptides that stimulate gluten-restricted T cells [5,6]. However, besides the hypothesis that mTG might be harmful by deamidating gliadin peptides, there are also hints that it is able to mask immunogenic gliadin epitopes by crosslinking [37,38,39,40]. One can assume that mTG is rather not taken up in an active form via food products due to multiple processing steps including heat treatment. However, one could speculate that its usage might cause newly formed immunogenic peptides that are consequently ingested and might represent neo-epitopes for the intestinal immune system [10].

In conclusion, we showed that the presence of the endogenous TG2 inhibitor ERp57 differs within the ER of duodenal biopsies of CD and control patients. This indicates a potential role of this protein in CD, potentially by inhibiting TG2 activity within the ER and preventing cross presentation of immunogenic gliadin peptides in healthy individuals. Our functional transglutaminase assay revealed PX12 and L-cystine as potent TG2 inhibitors using an oxidative mechanism. Both substances nearly abolished TG2 activity at micromolar doses and appeared to be much more potent than the competitive inhibitor ERW1041 in this setting. However, further research is clearly needed to investigate the effect of those molecules in cell culture and in vivo. Lastly, we could demonstrate that mTG activity is less regulated than TG2 and that common competitive TG2 inhibitors do not affect mTG. Since unregulated enzyme activity might increase the intestinal load of immunogenic peptides, additional effort is needed to clarify its role within food products and the intestinal microbiota.

## 4. Materials and Methods

### 4.1. Patients’ Characteristics and Preparation of Biopsies

This study was approved by the local ethics committee (reference number 119/16) and written informed consent was given from every patient. Excess duodenal biopsies were obtained during clinically indicated upper gastrointestinal endoscopy. At the time of endoscopy, all patients were on gluten-containing diet. Tissue samples from 5 CD (14 (9–15) years, 4 females) and 5 non-celiac disease patients (NCD, 12 (7–13) years, 3 females) were obtained. Diagnosis of CD was made according to current guidelines of the European Society for Paediatric Gastroenterology, Hepatology and Nutrition (ESPGHAN) [41,42]. All CD patients showed MARSH III lesions as well as positive anti-TG2-IgA antibodies (median 200, interquartile range 180 IU/mL). In the NCD group, 3 patients suffered from gastritis, 1 from eosinophilic esophagitis and 1 did not show any endoscopic, or histologic pathologies. None of the NCD patients had positive anti-TG2-IgA antibodies, or histological signs of duodenal inflammation. Samples were fixed in 5% paraformaldehyde-PIPES for 1h at room temperature, placed in polyvinyl-pyrrolidone sucrose at 4 °C overnight and frozen in liquid nitrogen. 

### 4.2. Electron Microscopy on Ultrathin Frozen Sections

Duodenal biopsies were sectioned and labelled using the postembedding technique according to Tokuyasu [43] and Griffiths et al. [24]. ERp57 was detected on ultrathin frozen sections (55 nM) of biopsies using a polyclonal rabbit anti-human antibody (PA3-009, Thermo Fisher Scientific, Langenselbold, Germany). TG2 was detected on a subcellular level by a polyclonal rabbit anti-human antibody (Kan5, kindly provided by Dr T. Mothes, Institute of Laboratory Medicine, university hospital Leipzig, Germany). For double labelling, a monoclonal mouse antibody against the ER marker protein disulfide isomerase (PDI, clone 1D3, Enzo Life Sciences, Lörrach, Germany) was used. Visualization of binding sites of the primary antibodies was achieved by gold-conjugated goat sera against rabbit (12 nM) and mouse immunoglobulin G (6 nM, Jackson ImmunoResearch, Suffolk, UK). Contrasting was carried out using uranyl acetate and 2% methylcellulose. RACE (Rapid Antigen Uptake into the Cytosol of Enterocytes) were identified electron microscopically by distinct morphological features such as reduced microvilli, electron-lucent cytosol and marginal condensation of heterochromatin [44,45].

### 4.3. Quantification of Subcellular Localization of ERp57

Subcellular quantification was performed using a transmission electron microscope (Philips EM 400 T, Kassel, Germany) by meander-shaped counting of 100 gold particles (12 nm) within the epithelium. Specificity of ERp57-antibody was assessed by evaluating the labelling of goblet cell granules as negative control. For quantifying the intracellular distribution of ERp57 within the epithelium, enterocytes and RACE were screened in a meander-shaped manner and 100 gold particles (12 nm) were counted. To investigate the concentration of ERp57 in the ER, 10 standardized images (magnification × 17,700) of enterocytes and RACE of each patient were taken. To evaluate the ER volume and the concentration of ERp57 within this compartment, a standardized grid (square side length: 4000 µm) and the following formulas were used to calculate organelle circumference (OC) and labelling density (LD) as adapted from Griffiths [24].

OC (µm) = 1/(ISm * M / ISlu *(1)

LD (GP/µm²) = G * M^2^ / ISlu * GF^2^ (2)

G = number of gold particles, GF = Grid factor (4,000), Ism = Intersections ER membrane, ISlu = Intersections ER lumen, M = Magnification (× 24,000).

### 4.4. In Vitro Transamidation Activity of TG2 and mTG

Evaluation of in vitro transamidation activity of both transglutaminases was performed according to Yi et al. [11]. In brief, tissue-culture treated 96-well plates were incubated with TG2 (T022, Zedira, Darmstadt, Germany), or mTG (T001, Zedira) for 30 min at 37 °C following the application of inhibitors and enhancers for 30 min. After washing, 200 µM of the substrate 5BP (5-(biotinamido)pentylamine) was applied in Tris-EDTA buffer with 5 mM CaCl_2_ (pH 7.5) for 3 h. After fixation with PFA 4% and blocking overnight with BSA 2%, streptavidin-HRP (1:2500, Biolegend, San Diego, CA, USA) was applied for 1 h. Finally, 100 µL per well of the substrate TMB (tetramethylbenzidine, Sigma-Aldrich, St. Louis, MO, USA) was added and the reaction was monitored continuously for 15 min using a microplate reader at 640 nm. The redox reagent dithiothreitol (DTT), which is commonly used to stabilize disulfide bonds within enzymes, was used as positive control. The molecular formula of mTG-blocker (C102, Zedira) was not disclosed by the manufacturer. All tested substances are listed in Table 2. Data were evaluated after background subtraction was performed against conditions where no transglutaminases were applied. Data were normalized against a control condition where transglutaminases were not treated with inhibitors. Experiments were at least performed in triplicates with three technical replicates per condition.

### 4.5. Statistics

Statistical analysis was performed using SPSS 21 (IBM, Chicago, IL, USA) and GraphPad Prism 9 (GraphPad Prism Software Inc, La Jolla, CA, USA). Students unpaired two-tailed *t* test (with Welch’s correction where appropriate), or Mann-Whitney U test were used where appropriate. Data are given as mean ± standard deviation (normally distributed data), or as median with interquartile range (IQR) (not normally distributed data).

## Figures and Tables

**Table 1:** Intracellular distribution of ERp57 in enterocytes and RACE.

	Enterocytes	RACE	Statistics
**labels per cell**	median: 10, IQR 4	median: 18, IQR 40	*p* < 0.001
**surface of the ER**	median: 0.7, IQR 0.4 µm	median 4.6, IQR 3 µm	*p* < 0.001
**apical membrane**	median: 5, IQR 4%	median: 1, IQR 3%	*p* < 0.01
**vacuoles**	mean: 37 ± 11%	mean: 45 ± 10%	*p* = 0.1
**endoplasmic reticulum**	mean: 18 ± 4%	mean: 30 ± 7%	*p* < 0.001
**basal membrane**	median: 10, IQR 28%	median: 0, IQR 11%	*p* < 0.01
**cytosol**	mean: 23 ± 6%	mean: 15 ± 7%	*p* < 0.01

**Table 2:** List of the tested endogenous and exogenous substances for the in vitro assay.

Substance	Effect	Company
**DTT (Dithiothreitol)**	Stimulation/reduction	Sigma-Aldrich, St. Louis, MO, USA
**TRX**	Stimulation/reduction	Abcam, Cambridge, UK
**L-cystine**	Inhibition/oxidation	Sigma-Aldrich, St. Louis, MO, USA
**ERp57**	Inhibition/oxidation	Novus Biologicals, Littleton, CO, USA
**ERW1041**	Inhibition/competition	Sigma-Aldrich, St. Louis, MO, USA
**mTG-blocker**	Inhibition/competition	Zedira, Darmstadt, Germany
**PX12**	Inhibition/oxidation	Sigma-Aldrich, St. Louis, MO, USA

ERp57, endoplasmic reticulum-resident protein 57; TRX, thioredoxin 1.

## Data Availability

The data presented in this study are available on request from the corresponding author.
